# Isolation and purification of *Eleutherococcus sessiliflorus* (Rupr. & Maxim.) S. Y. Hu peptides and study of their antioxidant effects and mechanisms

**DOI:** 10.3389/fphar.2024.1353871

**Published:** 2024-02-08

**Authors:** Chang Liu, Xuying Ding, Yining Xie, Chen Chen, Meijun Zhao, Yanming Duan, Guojing Yuan, Junxi Ren

**Affiliations:** ^1^ College of Pharmacy, Beihua University, Jilin, China; ^2^ Affiliated Hospital of Yanbian University, Yanji, China; ^3^ Department of Clinical Pharmacy, Affiliated Hospital of Jilin Medical College, Jilin, China

**Keywords:** *E. sessiliflorus*, peptides, oxidative stress, antioxidants, photoaging, matrix metalloproteinases

## Abstract

Oxidative stress is a state of imbalance between oxidant and antioxidant effects in the body, which is closely associated with aging and many diseases. Therefore, the development of antioxidants has become urgent. In this study, we isolated three polypeptides, G-6-Y, P-8-R, and F-10-W, from *Eleutherococcus sessiliflorus* (Rupr. & Maxim.) S. Y. Hu (*E. sessiliflorus*), based on the antioxidant and anti-aging properties of *Eleutherococcus*, and screened the most powerful free radical scavenging peptide P-8-R. Ultraviolet B (UVB)-induced oxidative stress damage in the skin was established to test the efficacy of P-8-R. In cellular experiments, P-8-R not only prevented oxidative stress damage in HaCaT cells, reduced intracellular reactive oxygen species levels, and inhibited the overexpression of matrix metalloproteinases but also inhibited apoptosis via the mitochondria-dependent apoptotic pathway; in animal experiments, P-8-R was able to prevent oxidative stress damage in the skin and reduce skin collagen loss by inhibiting the overexpression of MMPs to prevent mouse skin aging. In conclusion, the present study contributes to an in-depth understanding of the active compounds of *Eleutherococcus*, which is of great significance for the pharmacodynamic mechanism and industrial development of *Eleutherococcus*, and P-8-R is likely to become a potential antioxidant and anti-aging drug or skin care cosmetic in the future.

## 1 Introduction

Reactive oxygen species (ROS) are normal metabolites generated during cellular oxidative respiration, but are extremely oxidatively active and can undergo peroxidation reactions with intracellular nucleic acids, proteins, and membrane lipids, thereby causing cellular structural damage and metabolic disorders (Hauck et al., 2019; Jakubczyk et al., 2020). The antioxidant system in the human body is capable of scavenging ROS *in vivo*, and key antioxidant enzymes play an important role in maintaining redox balance in the body. The antioxidant system can be overwhelmed, leading to the development of oxidative stress, which in turn is associated with the onset and progression of a variety of diseases, including neurodegenerative diseases, atherosclerosis, hypertension, ischemia–reperfusion injury, and photoaging (Guzik and Touyz, 2017; Réus et al., 2019; Singh et al., 2019; Kibel et al., 2020; Liu et al., 2022). Therefore, effective scavenging of ROS is the key to the prevention and control of oxidative stress-related diseases.

*Eleutherococcus sessiliflorus* (Rupr. & Maxim.) S. Y. Hu (*E. sessiliflorus)* is one of the most common species of the genus *Eleutherococcus*. It is a local traditional Chinese medicine in the Changbai Mountain area of Jilin Province, China, and its root bark, called Wuga bark, shows efficacy in delaying aging, dispersing wind-dampness, and tonifying the liver and kidneys in many ways (Choi et al., 2021a; Choi et al., 2021b). Modern pharmacological studies have shown that the active constituents of *E. sessiliflorus* have a variety of pharmacological activities such as immunomodulation (Kim et al., 2013) and treatment of cardiovascular diseases (Wang et al., 2021) and are anti-inflammatory (Kim et al., 2020), sedative (Song et al., 2017), anti-tumor (Thamizhiniyan et al., 2015), and antioxidant (Chen et al., 2020). Therefore, *E.* sessi*liflorus* has prospects such as social value and economic benefits, both as a medicine and a healthcare functional food. A large number of studies have found that various components in *E. sessiliflorus* have antioxidant effects, such as polysaccharides, total flavonoids, and extracts (Adamczyk et al., 2019; Ganogpichayagrai and Suksaard, 2020), and studies have also shown that *E. sessiliflorus* is rich in amino acid compounds. Researchers have detected 16 kinds of amino acids in *E. sessiliflorus*, and polypeptides from *E. sessiliflorus* may have the same function of being antioxidant and anti-aging, which is worthy of in-depth study.

Peptide is a kind of material with a molecular weight between that of amino acids and proteins in the body. It is a kind of active compound needed by the organism to complete various physiological activities, and it plays a role in biological metabolic activities and disease treatment. Peptides not only play an essential role in biological functions but are also non-toxic, efficient, easy to absorb, cheap, and easy to obtain. Therefore, active peptides have made great achievements in food and cosmetics research and in other fields, for example, sea cucumber polypeptide (Lu et al., 2022), *Agaricus blazei* polypeptide (Feng et al., 2020), rice-derived peptide (Liu et al., 2020), and *Olivancillaria hiatula* peptide (Gasu et al., 2018). Thus, *E. sessiliflorus* peptides with antioxidant functions may be novel drugs or health products for antioxidants.

In this study, based on the antioxidant and anti-aging properties of *E. sessiliflorus*, we isolated three peptides, F-10-W, P-8-R, and G-6-Y, from *E. sessiliflorus* by alkaline extraction and acid precipitation, proteolytic enzyme hydrolysis, and gel filtration chromatography. We then screened the peptide P-8-R as having the strongest free radical scavenging ability using chemical antioxidant assays such as DPPH, ABTS, and PTIO. Furthermore, a UVB-induced skin oxidative stress injury model was further established to investigate the antioxidant effect of the active peptide P-8-R and its mechanism, which provides a basis for the pharmaceutical application of P-8-R and lays a foundation for the full exploitation of the active compounds of *E. sessiliflorus* and its industrial development in the future.

## 2 Materials and methods

### 2.1 Chemicals

The dried root bark of *E. sessiliflorus* (Wuga bark) was purchased from Linding Herbal Plantation Ltd. (China); DPPH, ABTS, and PTIO reagents were obtained from MACKLIN (China); thiazolyl blue tetrazolium bromide (MTT), 3,3′-diaminobenzidine (DAB), and DCFH-DA were purchased from Sigma-Aldrich (United States); DMEM, fetal bovine serum (FBS), and 0.25% trypsin were purchased from Gibco (United States); DAPI and TUNEL kits were obtained from Beyotime (China); hydroxyproline (HYP) assay kits were purchased from Nanjing Jiancheng (China); neutral protease (50,000 U/g), RIPA lysate, H&E and Masson staining kits, MMP-1 and MMP-9 ELISA kits, and hydrogen peroxide (H_2_O_2_) were obtained from Solarbio (China); and all other chemicals were obtained from Beijing Chemical Factory (China).

### 2.2 Isolation and purification of peptides

The dried root barks of *E. sessiliflorus* (Wuga bark) were ground into dried defatted *Eleutherococcus* powder; the crude protein in *Eleutherococcus* was extracted by alkaline extraction and acid precipitation; the enzyme conditions were as follows: neutral protease, pH 7.0, an enzyme time of 2 h, an enzyme temperature of 45°C, a liquid–solid ratio of 120:1, and an enzyme additive quantity of 3200 U/g so as to obtain the protein enzyme solution of *E. sessiliflorus*; The peptides of different molecular weights were separated and purified by filtration chromatography on Sephadex G-25 and an AKTA protein purification chromatograph (GE-100, United States), and the molecular weights of the peptides were initially determined by tricine-SDS-PAGE gel analysis.

### 2.3 Peptide identification and synthesis

The precise molecular weight and amino acid sequence of the peptide were identified by LC-MS/MS; the *E. sessiliflorus* peptide with the identified amino acid sequence was synthesized by Dangang Biotechnology Co., Ltd. (China).

### 2.4 DPPH, ABTS, and PTIO detection

We analyzed the free radical scavenging ability of each peptide using chemical antioxidant assays like DPPH, ABTS, and PTIO. Briefly, the DPPH solution was added to the peptides for 30 min at room temperature (RT), the OD value was measured at 519 nm, and the radical scavenging rate of DPPH was calculated; the ABTS solution was added to the peptides for 10 min at RT, the OD value was measured at 734 nm, and the radical scavenging rate of ABTS was calculated; the PTIO solution was added to the peptides for 24 h at 37°C, the OD value was measured at 565 nm, and the radical scavenging rate of PTIO was calculated.

### 2.5 Analysis of ferrous ion chelation ability

To the sample, 250 μM FeCl_2_ was added. The mixture was placed in a water bath at 50°C for 30 min, and 500 μM phenanthrozine solution was added. The mixture was left to stand at RT for 5 min. The OD value was measured at 562 nm, and the ferrous ion chelation rate was calculated.

### 2.6 Cell culture and treatment

Human immortalized keratinocytes (HaCaT) were obtained from NewgainBio (Cat. No. CH1031), and the cells were cultured in DMEM with 10% FBS in a 5% CO_2_ incubator at 37°C. We first determined the optimal dose of P-8-R in the pre-test and then divided the experimental groups into four groups: control, P-8-R, UVB, and UVB + P-8-R. Control: cells were not exposed to UVB; P-8-R: cells were not exposed to UVB and treated with 0.2 μg/mL P-8-R for 24 h; UVB: as mentioned below; UVB + P-8-R: cells were pretreated with 0.2 μg/mL P-8-R for 1 h and then treated with P-8-R for 24 h after UVB radiation.

### 2.7 UVB cell damage modeling

We slightly modified the UVB modeling method (Ding et al., 2023). Briefly, we used UVB lamps (PL-S 9W; Royal Dutch Philips Electronics Ltd., Holland, wavelength range: 300–320 nm; peak value: 311 nm) and a UV intensity detector (TM213; Tenmars Electronics Ltd., Taiwan). For UVB irradiation, the DMEM in the cell plate was discarded and PBS (pH 7.3) was added, and the cell plate was placed at a vertical distance of 10 cm from the UV light source for irradiation with a power of 500 μW/cm^2^, an irradiation time of 60 s, and a final intensity of 30 mJ/cm^2^. After UVB irradiation, DMEM was added again, and the sample was incubated for 24 h.

### 2.8 Cell viability assay

Cell viability was determined by the MTT reduction assay. Briefly, after the treatment period, the cells were incubated with 0.5 mg/mL MTT for 4 h. The formazan precipitate was dissolved in 200 μL DMSO, and the absorbance at 570 nm was detected by using a microplate reader (BioTek, United States). The cell viability was expressed as a percentage of the control.

### 2.9 Morphological features

HaCaT cells were seeded in 6-well plates and incubated for 24 h at 37°C with 5% CO_2_. After the treatment period, the HaCaT cell morphology was observed by using an inverted optical microscope (Olympus, Japan).

### 2.10 Intracellular ROS measurement

The DCFH-DA assay was used to quantify ROS production (Liu et al., 2023). Briefly, after the treatment period, the DCFH-DA was added to the treated plates at a final concentration of 10 μM, which were then incubated in the dark at 37°C for 30 min. Fluorescence images were obtained by using an upright fluorescence microscope (Olympus, Japan), and relative fluorescence was measured using ImageJ 1.6 software.

### 2.11 Comet assay

The comet assay was used to analyze the recombinant protein for UVB-induced DNA damage (Chaiprasongsuk et al., 2019). Briefly, after placing the first layer of the HaCaT cell suspension, 0.7% agarose was added and solidified at 4°C for 10 min. The slides were then placed in the pre-cooled lysis buffer, lysed at 4°C for 2 h, and electrophoresed for 20 min. The slides were then removed and placed in PBS to neutralize the strong base. Each slide was stained with EB and then observed under a fluorescence microscope. The percentage of DNA content in the comet tail was analyzed using the CASP software. Tail DNA % was used as an index of DNA damage (Duan et al., 2019).

### 2.12 MMP-1 and MMP-9 ELISA assay

The levels of MMP-1 and MMP-9 in the supernatant of HaCaT cells were measured using MMP-1 and MMP-9 ELISA kits (Cat. No. SEKH-0251 and SEKH-0257; Solarbio). Briefly, after the treatment period, the supernatant was collected and centrifuged at 3,000 g for 10 min to discard the precipitate. The standard curves of MMP-1 and MMP-9 were then plotted, and the absorbance of the supernatant was detected at 450 nm by using a microplate reader (BioTek, United States).

### 2.13 Immunofluorescence (IF) staining

IF staining was used to detect the specific expression of MMP-1 and MMP-9 in the cells. We treated HaCaT cells with 4% paraformaldehyde fixation, 0.2% Triton X-100 permeabilization, and 1% BSA closure and finally added the following primary antibodies: MMP-1 (1:200; Cat. No. AF0231; Beyotime) and MMP-9 (1:200; Cat. No. K001664P; Solarbio) and then incubated the cells overnight at 4°C. The following day, goat anti-rabbit IgG-FITC secondary antibody (1:500; Cat. No. A0562; Beyotime) was added, and finally the nuclei were stained with DAPI, and images were obtained.

### 2.14 Animal treatment

This animal experiment was approved by the Laboratory Animal Ethics Committee of Beihua University. BALB/c mice (male, weight: 20–25 g, n = 60) were purchased from Changchun Yisi Laboratory Animal Technology Co. All mice were kept in a temperature-controlled environment with a 12-h light/dark cycle with free access to water and food. After 1 week, the mice were depilated on their backs over an area of 6 cm^2^ until the skin was completely exposed. The optimal dose of P-8-R was determined to be 0.1 mg/cm^2^ by a pre-test. After that, the mice were randomly divided into four groups. In the control group, saline was applied directly without UVB irradiation. In the P-8-R group, only 0.1 mg/cm^2^ P-8-R was applied. In the UVB group, the UVB modeling method was slightly modified (Divya et al., 2015). Briefly, the backs of the mice were irradiated at a vertical distance of 5 cm from the UVB source with an irradiation power of 1,000 μW/cm^2^ and a final intensity of 120 mJ/cm^2^ three times per week for 4 weeks. For the UVB + P-8-R group, 0.1 mg/cm^2^ P-8-R was injected into the backs of the mice after 1 week, 2 h after each UVB irradiation. At the end of the experiment, all mice were euthanized, and the dorsal skin tissues were excised for various experiments.

### 2.15 Skin H_2_O_2_ and HYP tests

The amounts of H_2_O_2_ and HYP in the skin are measured using the appropriate kits [(Cat. No. A030-2-1; Solarbio) and (Cat. No. A030-2-1; Nanjing Jiancheng), respectively]. Briefly, the supernatant from the centrifugation of the skin tissue homogenate is reacted with appropriate reagents. Finally, the absorbance of the reactants at the corresponding wavelength is measured with a microplate reader (BioTek, United States), and the levels are calculated.

### 2.16 Pathological analysis

The skin tissue from the back of mice was fixed in 4% paraformaldehyde, dehydrated through a graded ethanol series, cleared in xylene, embedded in paraffin, and sectioned at 5 μm by using a microtome (Leica, Germany). Tissue sections were stained with hematoxylin and eosin (H&E) for epidermal hyperplasia studies and with Masson’s stain for collagen fiber analysis. All staining procedures were performed according to their respective staining schemes without modification. Histopathological changes were examined under a light microscope (Olympus, Japan).

### 2.17 TUNEL detection

TUNEL staining of the skin was carried out using an *in situ* cell death detection kit (Cat. No. C1091; Beyotime). Briefly, the sections were digested with proteinase K at 37°C for 20 min, and then the TUNEL reaction mixture was incubated at 37°C in the dark. The sections were then coverslipped with Converter-POD at 37°C for 30 min and then incubated with DAB for 3 min to observe the reaction. Finally, the sections were counterstained with hematoxylin for 30 s. The staining result was expressed as TUNEL-positive.

### 2.18 Immunohistochemistry (IHC)

We used IHC to detect the specific expression of MMP-1 and MMP-9 in skin tissues. After skin sections were deparaffinized, hydrated, cleared of endogenous peroxidase, antigenically repaired, and closed with 5% BSA, MMP-1 and MMP-9 primary antibodies were added and incubated overnight at 4°C in the dark. Goat anti-rabbit IgG-HRP (1:300; Cat. No. SE134; Solarbio) was added the following day, followed by DAB staining, hematoxylin re-staining, and differentiation. Finally, the sections were observed and photographed using an orthogonal microscope.

### 2.19 Western blotting

After treatment, HaCaT cells and skin tissue were extracted using RIPA lysis solution (Cat. No. P00138; Beyotime) containing 1% PMSF to extract proteins and quantified using BCA protein kits (Cat. No. PC0020; Solarbio). Protein samples (35 μg) were separated by SDS-PAGE using A 12% resolving gel and then transferred to PVDF membranes (Millipore, United States). The PVDF membranes were blocked for 1 h in PBS containing 1% BSA, and the PVDF membrane was incubated overnight at 4°C with primary antibodies, including MMP-1, MMP-9, Bax (1:1,000; Cat. No. 2772; CST), Bcl-2 (1:1,500; Cat. No. ab196495; Abcam), cleaved caspase-3 (1:1,000; Cat. No. 9661; CST), and β-actin (1:5,000; Cat. No. AF5003; Beyotime). The membranes were then incubated with the appropriate goat anti-rabbit secondary antibodies for 1 h at RT, followed by three washes in TBST. Proteins were detected using the ECL reagent. The loading protein was normalized to β-actin, and the target protein were exposed using a high-sensitivity chemiluminescence imaging system (Bio-Rad, United States).

### 2.20 Statistical analysis

Data were expressed as mean ± SD and were statistically analyzed with Prism software (GraphPad Software Inc., United States) and analyzed with ANOVA for single-factor analysis. The Student’s t-test was used for inter-group comparison. *p* < 0.05 was considered significant.

## 3 Results

### 3.1 Analysis of the ability of P-8-R to scavenge free radicals

**FIGURE 1:**
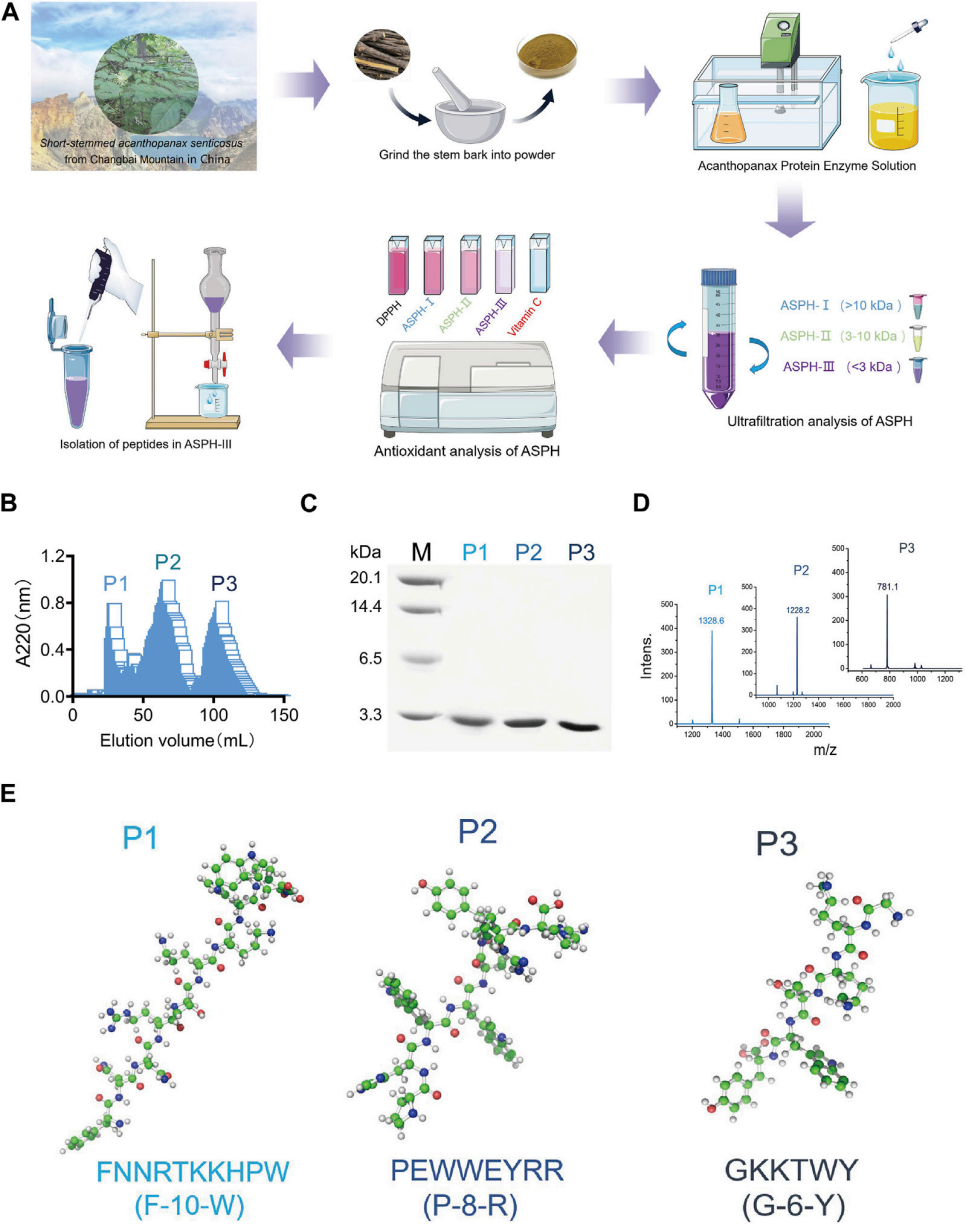
Isolation of active polypeptides of *Eleutherococcus sessiliflorus*. **(A)**. Flowchart for the isolation and purification of peptides from *Eleutherococcus sessiliflorus*. **(B)**. Sephadex G-25 elution enzyme digest curve. **(C)**. Tricine-SDS-PAGE gel analysis of different components. **(D)**. MALDI-TOF MS analysis of different components. **(E)**. Amino acid sequence and 3D structure of different components.

**FIGURE 2:**
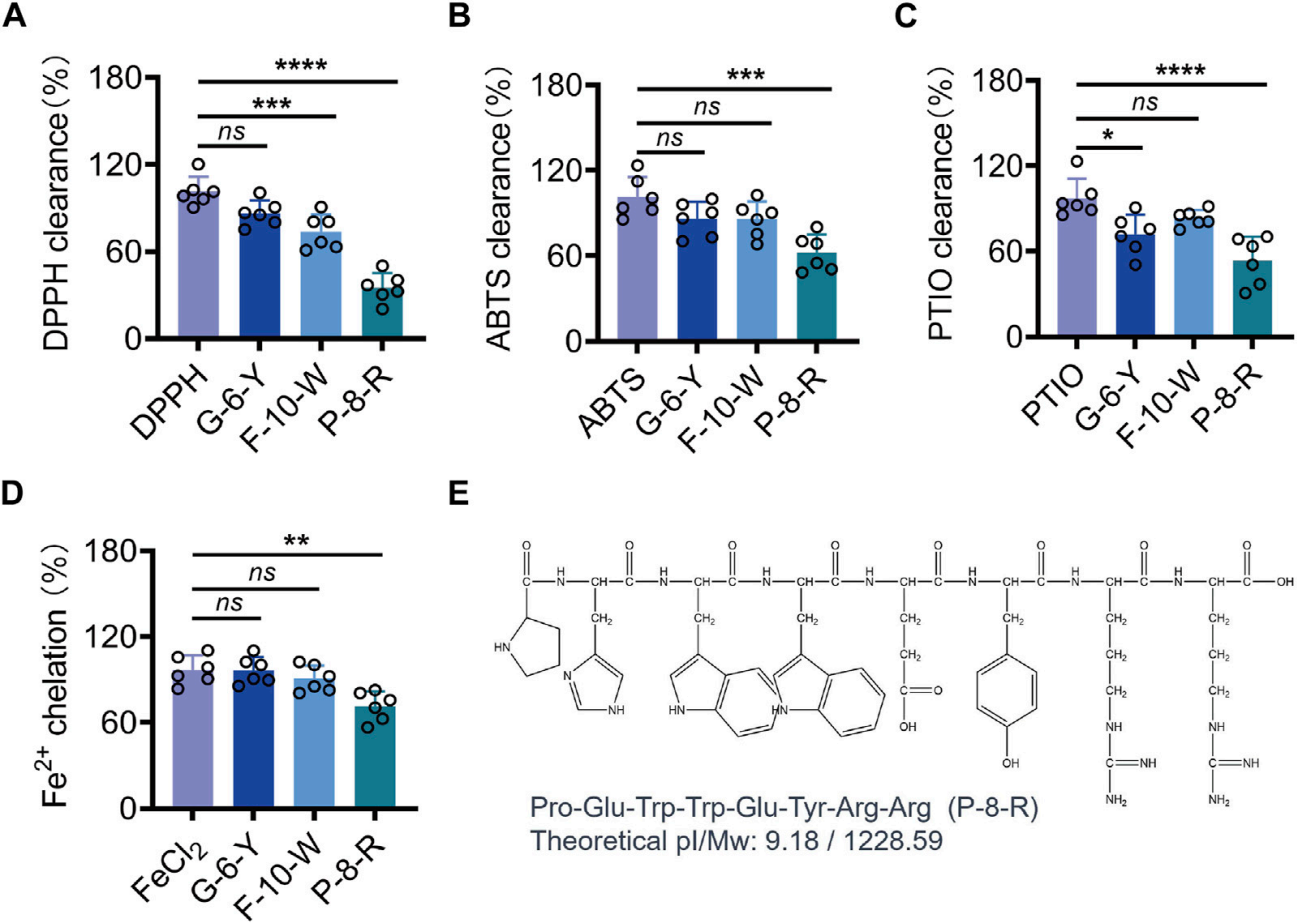
Analysis of DPPH, ABTS, PTIO, and the metal chelating ability of the three peptides. **(A)**. DPPH clearance rate. **(B)**. ABTS clearance rate **(C)**. PTIO clearance rate. **(D)**. Fe^2+^ chelation rate of the three peptides. **(E)**. Chemical formula of P-8-R. *ns* means no statistical significance (^*^ *p* < 0.05, ^**^ *p* < 0.01, ^***^ *p* < 0.001, and ^****^ *p* < 0.0001).

We purified three peptides, P1, P2, and P3 (Figure 1B), from the hydrolysate of *E. sessiliflorus* neutral protease using Sephadex G-25. Tricine-SDS-PAGE and MS analyses showed that the molecular weights of P1, P2, and P3 were 1,327.5 Da, 1,228.6 Da, and 781.9 Da, respectively. The amino acid sequences of P1, P2, and P3 were P1: FNNRTKKHPW (F-10-W), P2: PHWWEYRR (P-8-R), and P3: GKKTWY (G-6-Y), respectively (Figure 1C,E). In addition, we evaluated the ability of three peptides to scavenge oxygen and nitrogen radicals by the *in vitro* chemical antioxidant assay (DPPH, ABTS, and PTIO), and the experimental results are shown in Figures 2A–D. P-8-R has the strongest ability to scavenge oxygen and nitrogen radicals and can be used for subsequent antioxidant pharmacological studies.

### 3.2 P-8-R inhibits oxidative stress damage and apoptosis in HaCaT cells

**FIGURE 3:**
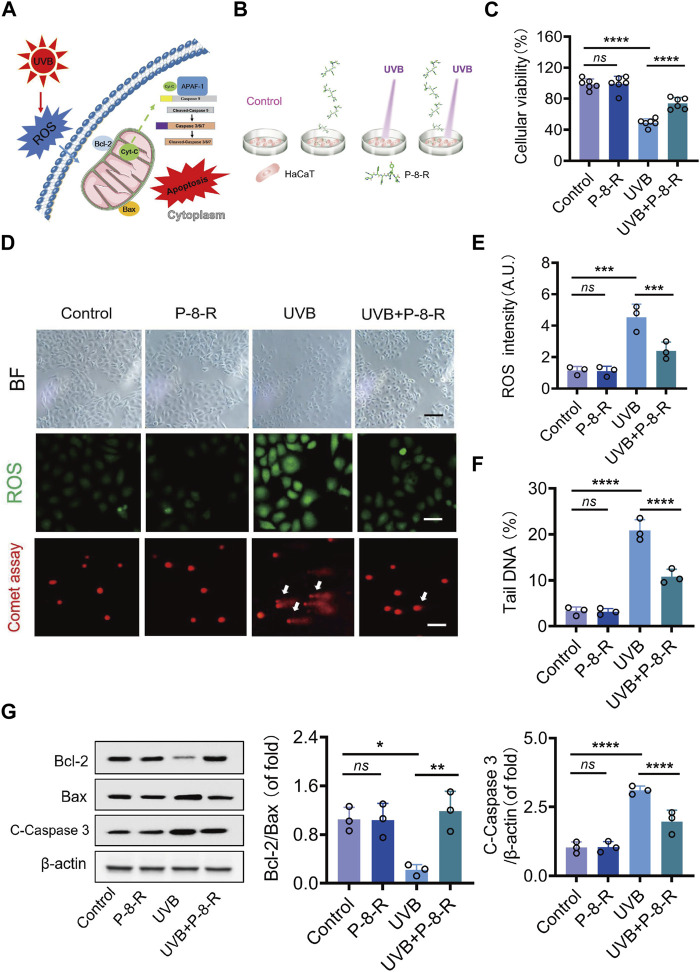
P-8-R inhibits HaCaT cell apoptosis via a mitochondria-dependent pathway. **(A)**. Schematic representation of the mitochondria-dependent pathway. **(B)**. Cellular experimental protocols. **(C)**. Cell viability. **(D)**. Representative images of brightfield microscopy, DCFH-DA staining, and comet assay. **(E)**. ROS intensity. **(F)**. The tail DNA rate of the comet assay in HaCaT cells. **(G)** Western blotting images and expression rates of Bcl-2, Bax, cleaved caspase-3, and β-actin in the HaCaT cells. Scale bar: 50 μm. *ns* means no statistical significance (^*^ *p* < 0.05, ^**^ *p* < 0.01, ^***^ *p* < 0.001, and ^****^ *p* < 0.0001).

To investigate the antioxidant effect of P-8-R, we established a UVB-induced HaCaT cell model. The MTT results are shown in Figure 3C. The cell viability of the cells in the UVB group was decreased, while P-8-R was able to significantly increase the cell viability. The results of cell morphology analysis showed that both the control and P-8-R groups had normal cell morphology without any abnormal changes; the cells in the UVB group showed morphological changes such as cell shrinkage, cell membrane folds, and cytoplasmic tightness, but the cell morphology in the UVB + P-8-R group did not change remarkably compared with that in the UVB group, which indicated that P-8-R had a strong cytoprotective effect. In order to study the mechanism of action of P-8-R, we examined the oxidative stress and apoptosis of HaCaT cells by DCFH-DA staining and comet assay, respectively, and the results are shown in Figure 3D. Compared with the control group, the fluorescence intensity of intracellular DCF in the UVB group was significantly enhanced, but P-8-R could obviously weaken the fluorescence intensity of intracellular DCF, which indicated that P-8-R could protect HaCaT cells from oxidative stress by scavenging ROS. After that, we detected the cellular DNA damage by comet assay, and the results showed that the trailing cell rate of cells in the UVB group increased with DNA breakage and structural damage, while the trailing cell rate of cells in the UVB + P-8-R group decreased, which indicated that P-8-R was able to inhibit UVB-induced DNA damage in HaCaT cells. Additionally, we detected the key proteins in the mitochondria-dependent apoptosis pathway by Western blotting, and the results are shown in Figure 3G. The protein expression rate of Bcl-2/Bax in cells of the UVB group was significantly decreased, and the protein expression of cleaved caspase-3 was remarkably elevated. However, P-8-R could increase the protein expression rate of Bcl-2/Bax and downregulate the protein expression of cleaved caspase-3, which indicated that P-8-R could inhibit HaCaT cell apoptosis through the mitochondria-dependent apoptosis pathway.

### 3.3 P-8-R decreases the expression and secretion of MMPs in HaCaT cells

**FIGURE 4:**
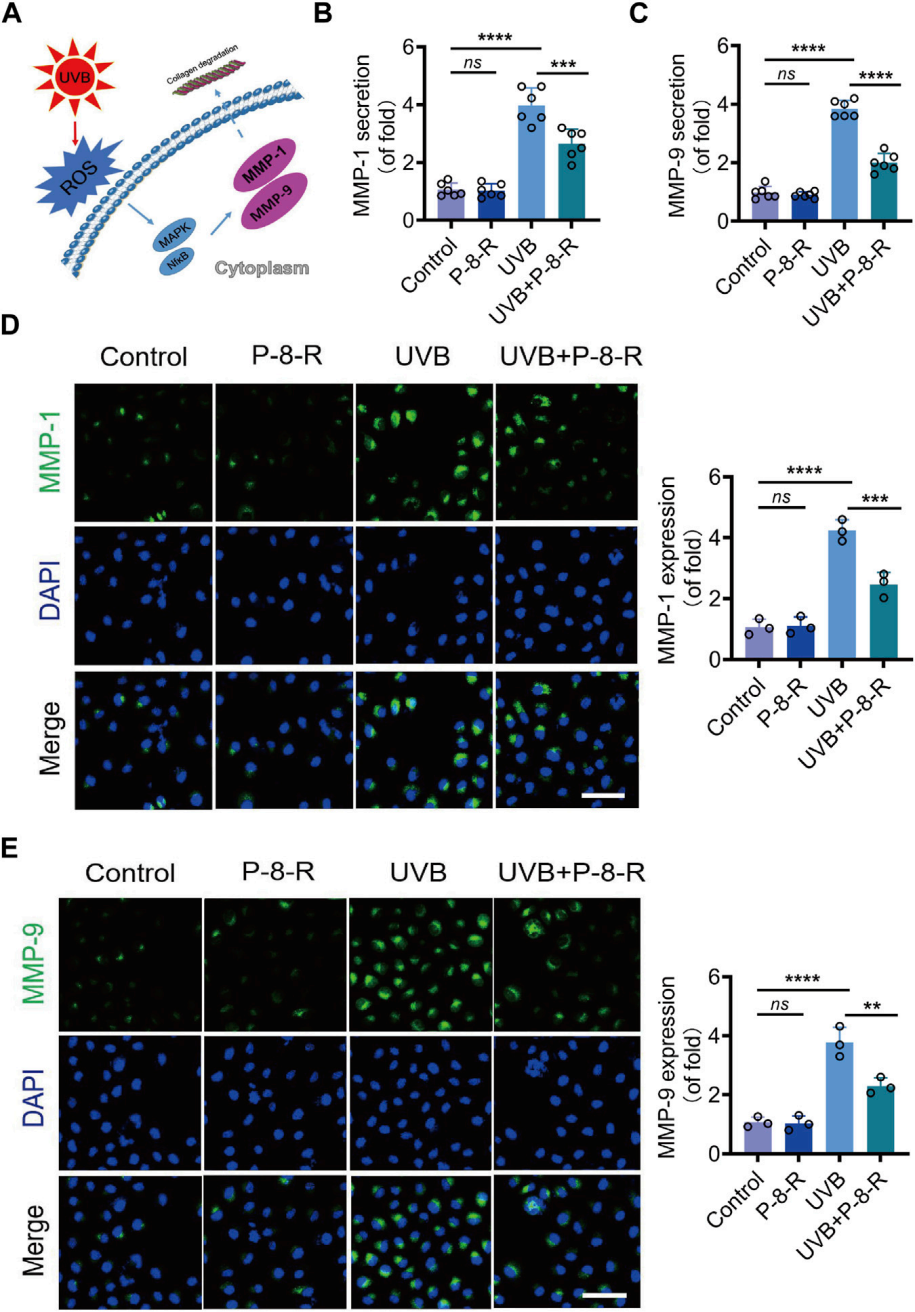
Effect of A/D-FLip on MMPs in HaCaT cells. **(A)** Schematic representation of UVB-induced MMPs. Extracellular secretion of **(B)** MMP-1 and **(C)** MMP-9. IF images and protein expression of **(D)** MMP-1 and **(E)** MMP-9. Scale bar: 50 μm. *ns* means no statistical significance (^**^ *p* < 0.01, ^***^ *p* < 0.001, and ^****^ *p* < 0.0001).

We examined the levels of MMP-1 and MMP-9 in the supernatants of HaCaT cells, and the results are shown in Figure 4. Compared with the control group, the levels of MMP-1 and MMP-9 in the cell supernatants of the UVB group were remarkably increased, whereas compared with the UVB group, the levels of MMP-1 and MMP-9 in the cell supernatants of the UVB + P-8-R group were notably decreased, indicating that P-8-R was able to inhibit UVB-induced MMP-1 and MMP-9 protein overexpression in skin epidermal cells and reduce the protein secretion of MMP-1 and MMP-9.

### 3.4 P-8-R ameliorates pathological injury in mouse skin

**FIGURE 5:**
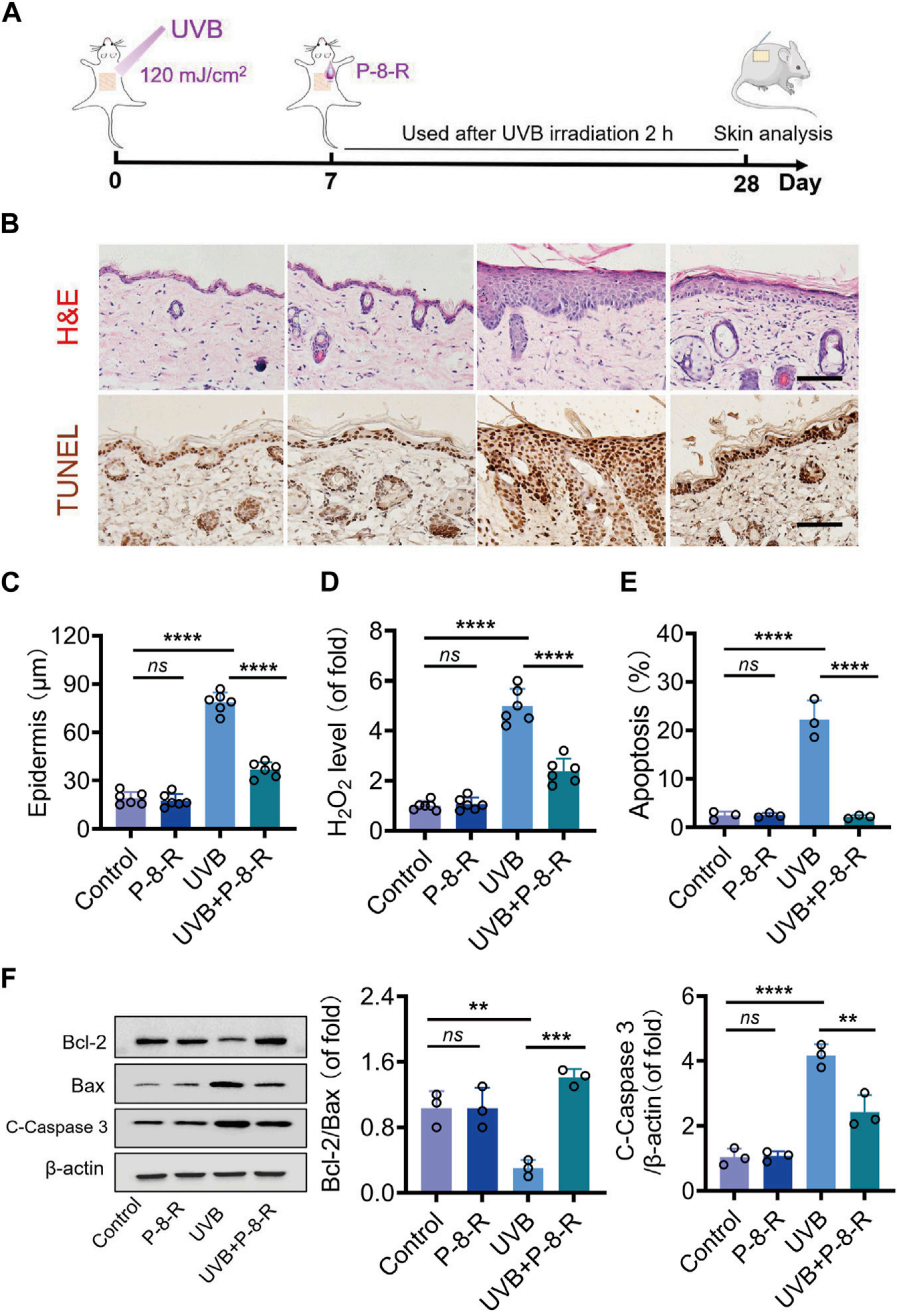
Effect of P-8-R on UVB-induced skin injury. **(A)**. Schematic diagram of the timeline of the animal experiment. **(B)**. H&E staining images of mouse skin. **(C)**. Skin epidermal thickness analysis (from H&E measurements). **(D)**. H_2_O_2_ content. **(E)** The apoptosis rate of skin cells. **(F)** Western blotting images and protein expression of Bcl-2, Bax, cleaved caspase-3, and β-actin in the skin. Scale bar: 100 μm. *ns* means no statistical significance (^**^ *p* < 0.01, ^***^ *p* < 0.001, and ^****^ *p* < 0.0001).

The results of H&E staining and epidermal thickness measurements are shown in Figure 5B, C. The skin structure of mice in the control and P-8-R groups was intact, and the stratum corneum, epidermis, and dermis were clearly visible, with clear epidermal–dermal boundaries. In the UVB group, the skin of mice was significantly thickened, and the epidermal–dermal boundary disappeared, indicating that the UVB-induced ROS were capable of contributing to pathological changes in the skin. The mice in the UVB + P-8-R group showed a decrease in the thickness of the skin and epidermis, and the epidermal–dermal boundary was clearer. To further study the damage caused by oxidative stress in the skin, we detected the level of H_2_O_2_ in the skin, and the results showed that P-8-R was able to remarkably reduce the level of H_2_O_2_ in the skin and prevent the damage caused by oxidative stress in the skin (Figure 5D). Moreover, TUNEL and Western blotting results showed that P-8-R could inhibit skin cell apoptosis through the mitochondria-dependent apoptotic pathway, as opposed to the *in vitro* experiments. In short, P-8-R could reduce the pathological changes caused by UVB-induced oxidative stress in mouse skin through antioxidant and anti-apoptotic effects.

### 3.5 P-8-R prevents collagen loss in mouse skin

**FIGURE 6:**
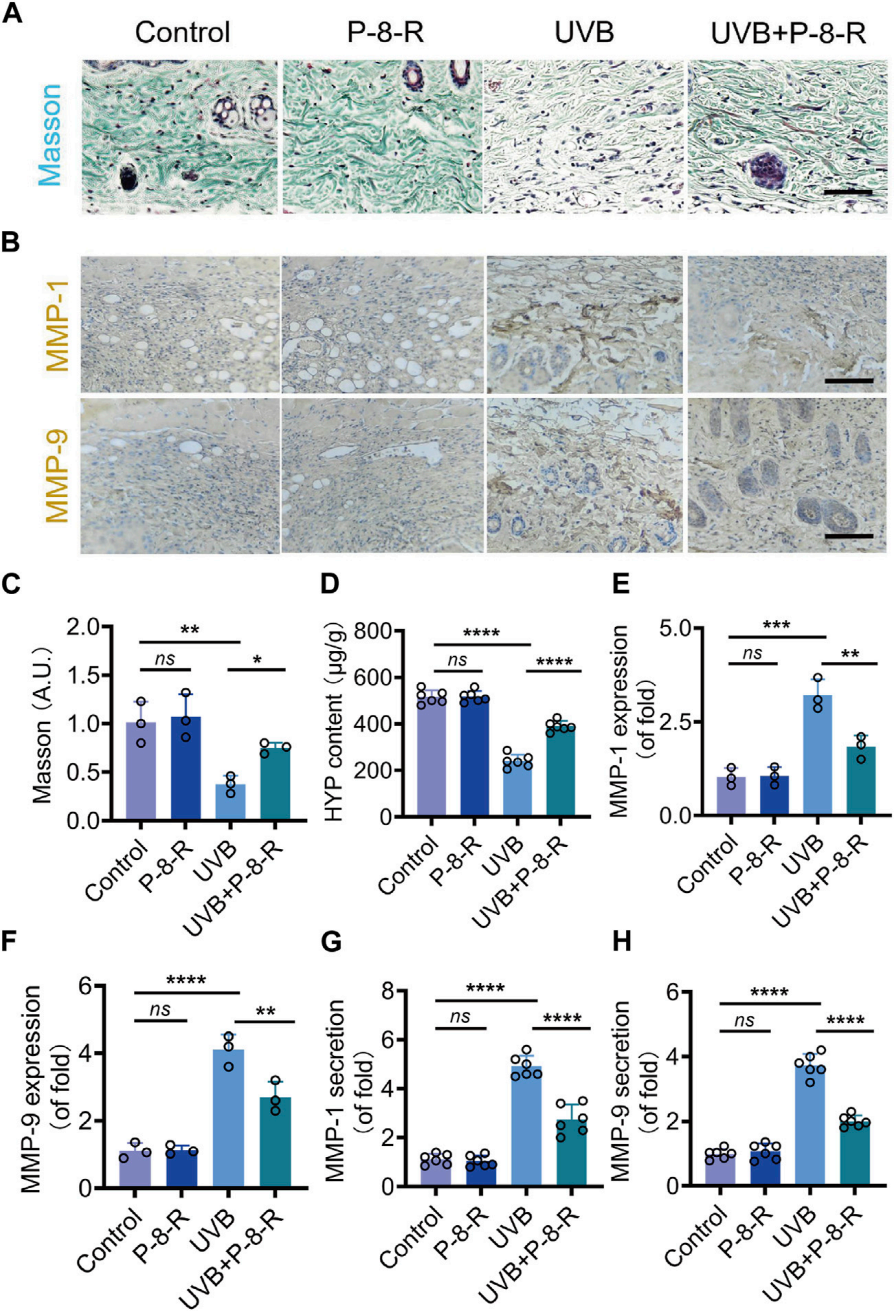
Effect of P-8-R on UVB-induced skin collagen loss. **(A)**. Masson staining images. **(B)**. Immunohistochemistry images of MMP-1 and MMP-9. **(C)**. Intensity of Masson’s stain. **(D)**. HYP content. Secretion of **(E)** MMP-1 and **(F)** MMP-9. Protein expression of **(G)** MMP-1 and **(H)** MMP-9 in the skin. Scale bar: 100 μm. *ns* means no statistical significance (^**^ *p* < 0.01, ^***^ *p* < 0.001, and ^****^ *p* < 0.0001).

**FIGURE 7:**
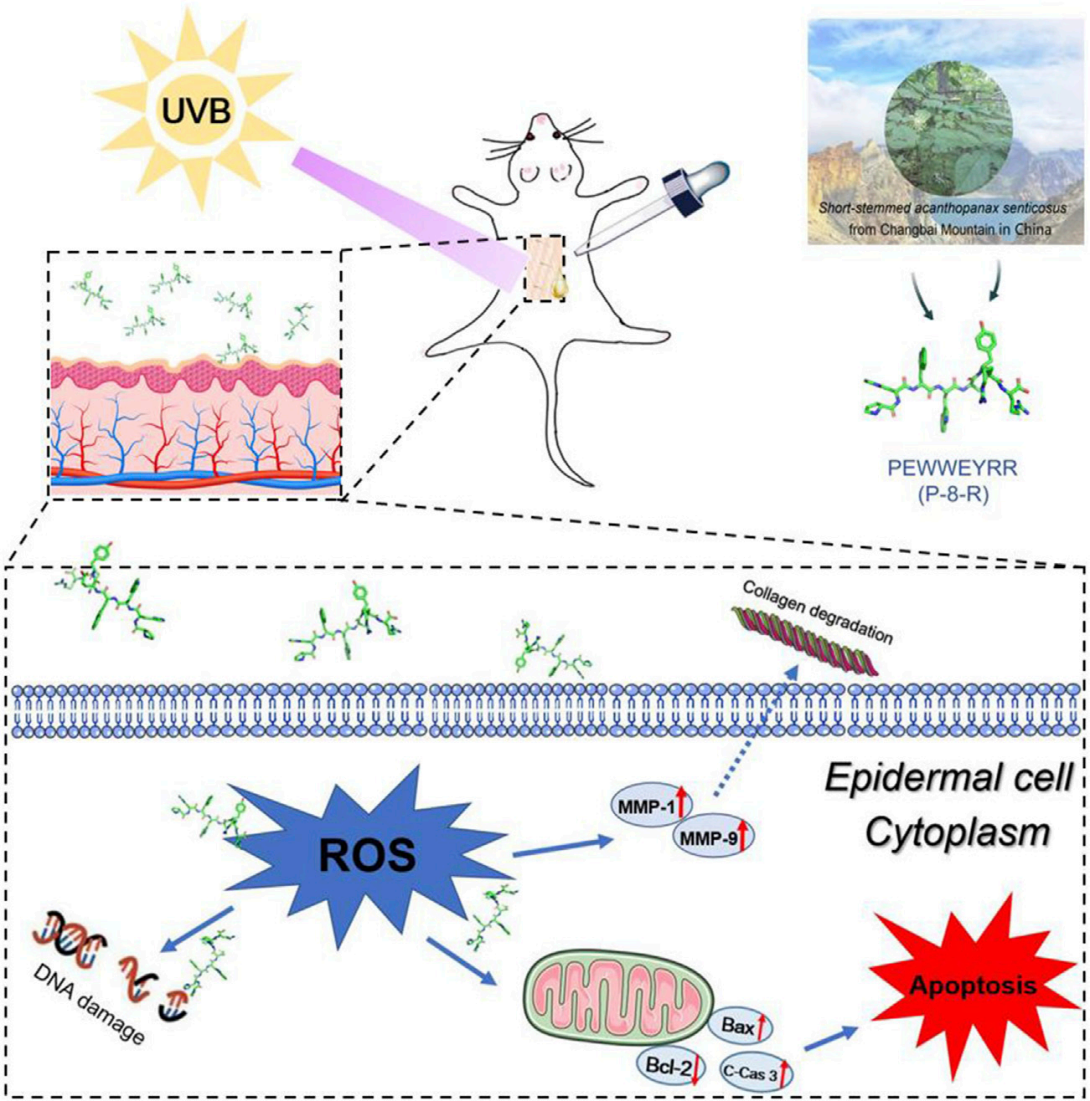
Schematic diagram of P-8-R inhibiting UVB-induced skin oxidative stress injury.

We detected the collagen density and content in the skin of mice by Masson staining as well as the HYP kit, and the results showed that P-8-R could keep the collagen density of the skin basically normal (Figure 6A–D) (Figure 7). We further examined the expression and secretion of MMP-1 and MMP-9 in the skin of mice. As shown in Figure 6E–H, P-8-R could notably downregulate the UVB-induced overexpression and secretion of MMP-1 and MMP-9, which suggests that P-8-R could suppress the overexpression of MMP-1 and MMP-9 proteins, thereby reducing the loss of collagen in mouse skin and preventing the skin from photoaging. All in all, P-8-R could inhibit UVB-induced oxidative stress in mouse skin and provide protection against aging caused by collagen loss.

## 4 Discussion

*E. sessiliflorus* is a traditional tonic Chinese herbal medicine from the Changbai Mountain region of China, with mild medicinal properties, low toxicity, and antioxidant, anti-aging, and anti-fatigue properties (Li et al., 2016; Jia et al., 2021; Li et al., 2022; Zhang and Zhu, 2022). *E. sessiliflorus* is rich in a variety of active ingredients, such as plant proteins, peptides, and polysaccharides, all of which have potent and strong antioxidant activity and hold promise for antioxidant drug development. Researchers use 75% methanol to obtain multiple components from *E. sessiliflorus*, which are mainly flavonoids, polyphenols, and phenolic acids with antioxidant and anti-acetylcholinesterase properties (Hauck et al., 2019). It is well-known that peptides have molecular weights ranging between those of proteins and amino acids and play important roles in life activities and disease treatment. Compared to small molecules, peptides have more advantages and unique functions, such as the ability to be absorbed preferentially, rapidly, and completely. Therefore, in this study, three polypeptides, G-6-Y, P-8-R, and F-10-W, were isolated and purified from the plant proteins of *E. sessiliflorus*, and the chemical antioxidant test results showed that P-8-R could not only effectively scavenge nitrogen and oxygen radicals, but also chelate iron ions in the solution, and that it possessed significant antioxidant ability compared with the methanol extract of *E. sessiliflorus*.

The amino acid composition and the arrangement of the peptide sequence directly affect the biological activity of the peptide. The amino acid sequence in P-8-R consists of Pro–Glu–Trp–Trp–Glu–Tyr–Arg–Arg, where Tyr is an aromatic amino acid, which is a hydrogen donor. The photo group on Tyr can provide a hydrogen atom for free radicals so that the free radicals can reach a stable state and the amino acids, after losing the hydrogen atoms, can form stable compounds through resonance, thus achieving the effect of scavenging free radicals (Wu et al., 2015). Proline is a hydrophobic amino acid that can improve the lipid solubility of polypeptides and facilitate the contact of polypeptides with lipid free radicals, thus facilitating the exertion of their lipid peroxidation inhibitory effects. Arg is a functional amino acid that plays an important role in physiological functions, metabolism, and nutrition in the body. Studies have shown that the guanidinium group in Arg can provide electrons to free radicals to bring them to a stable state and terminate the free radical chain reaction, thus exhibiting a strong reducing ability (Sun et al., 2023). Two Arg at the C-terminal end of P-8-R are positively charged, which is conducive to the binding of P-8-R to the biofilm, and the addition of other hydrophobic amino acids can enable P-8-R to penetrate through the stratum corneum of the skin and penetrate the cell membrane to enter the epidermal cells to exert the anti-photoaging effect. Moreover, there are a large number of free metal ions in living organisms that catalyze the generation of hydroxyl radicals from H_2_O_2_ via the Fenton reaction, causing oxidative damage to the skin (Rinnerthaler et al., 2015; Prasad et al., 2018). In this study, P-8-R was found to be able to chelate Fe^2+^, which may be related to the chelating effect of Trp and Tyr on free metal ions in the P-8-R sequence.

When the skin is irradiated with UVB, the skin chromophore transfers electrons to oxygen in its excited state, thereby generating a large amount of O^2.-^, which later leads to further overproduction of ROS and promotes oxidative stress damage and skin aging. In order to test the antioxidant effect of P-8-R, we established a model of UVB-induced oxidative stress damage in the skin. In the experiment, P-8-R could effectively scavenge ROS through the skin’s stratum corneum into the epidermal cells, preventing oxidative stress-induced damage to the skin’s epidermal cells. Additionally, mitochondria-dependent apoptosis is one of the major pathways of apoptosis, and we found that P-8-R could increase the expression rate of Bcl-2/Bax proteins and inhibit the expression of cleaved caspase-3 proteins. This suggests that P-8-R may inhibit epidermal cell apoptosis through a mitochondria-dependent pathway. In the extracellular matrix, MMP-1 is able to degrade type I and type III collagen, and MMP-1 is also able to degrade collagen together with MMP-9 and reduce the self-repair ability of damaged collagen (Kwon et al., 2011; Hwang et al., 2014; Lee et al., 2016). Therefore, ROS scavenging is important for maintaining normal collagen synthesis. In the study, P-8-R could also to indirectly inhibit the overexpression of MMP-1 and MMP-9 through the timely scavenging of ROS and maintain a basically normal level of collagen, thus preventing injury to the skin caused by oxidative stress and aging induced by excessive UV irradiation. All in all, P-8-R was a potential natural antioxidant peptide capable of preventing skin damage and having anti-aging effects (Figure 7).

## 5 Conclusion

In this study, we found that P-8-R polypeptide derived from *E. sessiliflorus* has a strong antioxidant effect. It not only prevents oxidative stress damage and apoptosis in skin cells but also inhibits skin collagen loss, thus preventing skin aging. The present study contributes to a deeper understanding of the effective material basis of *E. sessiliflorus*, which is of great significance for the pharmacodynamic mechanism and industrial development of *E. sessiliflorus*. Although the deeper anti-aging mechanism of P-8-R remains to be investigated, it is likely to be a potential antioxidant drug or skin care cosmetic in the future.

## Data Availability

The original contributions presented in the study are included in the article/Supplementary Material; further inquiries can be directed to the corresponding author.
